# The Antimicrobial Activity of the AGXX® Surface Coating Requires a Small Particle Size to Efficiently Kill *Staphylococcus aureus*

**DOI:** 10.3389/fmicb.2021.731564

**Published:** 2021-08-12

**Authors:** Nico Linzner, Haike Antelmann

**Affiliations:** Freie Universität Berlin, Institute for Biology-Microbiology, Berlin, Germany

**Keywords:** *Staphylococcus aureus*, AGXX®, metal particles, antimicrobial activity, contact killing

## Abstract

Methicillin-resistant *Staphylococcus aureus* (MRSA) isolates are often resistant to multiple antibiotics and pose a major health burden due to limited treatment options. The novel AGXX® surface coating exerts strong antimicrobial activity and successfully kills multi-resistant pathogens, including MRSA. The mode of action of AGXX® particles involves the generation of reactive oxygen species (ROS), which induce an oxidative and metal stress response, increased protein thiol-oxidations, protein aggregations, and an oxidized bacillithiol (BSH) redox state in *S. aureus*. In this work, we report that the AGXX® particle size determines the effective dose and time-course of *S. aureus* USA300JE2 killing. We found that the two charges AGXX®373 and AGXX®383 differ strongly in their effective concentrations and times required for microbial killing. While 20–40 μg/ml AGXX®373 of the smaller particle size of 1.5–2.5 μm resulted in >99.9% killing after 2 h, much higher amounts of 60–80 μg/ml AGXX®383 of the larger particle size of >3.2 μm led to a >99% killing of *S. aureus* USA300JE2 within 3 h. Smaller AGXX® particles have a higher surface/volume ratio and therefore higher antimicrobial activity to kill at lower concentrations in a shorter time period compared to the larger particles. Thus, in future preparations of AGXX® particles, the size of the particles should be kept at a minimum for maximal antimicrobial activity.

## Introduction

The increasing prevalence of antibiotic resistant strains in hospitals and the community poses a major burden to human health and limits treatment options of life-threatening bacterial infections (Christaki et al., 2020). Of particular importance are ESKAPE pathogens, such as multi-resistant *Staphylococcus aureus* isolates, which can cause severe systemic infections and acquire quickly new resistance elements through horizontal gene transfer (Mulani et al., 2019; De Oliveira et al., 2020). Promising alternative antimicrobial compounds could be reactive oxygen species (ROS) producing antimicrobials, which target rather non-specifically multiple cellular targets, such as proteins, lipids, and nucleic acids and therefore can eliminate drug-resistant bacteria (Lewis, 2013; Vatansever et al., 2013).

Metals, like silver (Ag^+^) and copper (Cu^2+^), have been used since ancient times for medication of bacterial infections and were shown to exert their microbicidal activity *via* ROS generation and protein and membrane damage (Lemire et al., 2013; Mijnendonckx et al., 2013). ROS generated from Ag^+^ has been shown to damage the bacterial cell envelope by disruption of the peptidoglycan cell wall, lipoteichoic acids, and the phosphatidylethanolamine membrane lipids (Gunawan et al., 2020). Furthermore, silver condensed the DNA and caused protein damage *via* its interaction with protein thiols, the release of Fe^2+^ from FeS clusters, or by mismetallation of Zn^2+^-containing proteins (Barras et al., 2018). However, the widespread use of silver in the treatment of wounds and burns has selected for various silver resistance mechanisms (Gupta et al., 1999; Silver, 2003; Atiyeh et al., 2007; Mijnendonckx et al., 2013). To circumvent the problem of silver resistance, silver nanoparticles were produced, which act as antimicrobials *via* the release of silver ions (Xiu et al., 2012). The application of silver ions and nanoparticles has raised concerns due to their toxic effects in eukaryotic cells (Mijnendonckx et al., 2013). Silver was shown to precipitate in various tissues and organs in the mice, including the kidney, liver, jejunum, colon, and the brain (Boudreau et al., 2016; Recordati et al., 2021). Neurotoxic effects of silver have been reported, including argyria, which are rare cases of irreversible gray pigmentations of the skin and the eyes due to silver sulfide precipitates (Lansdown, 2010; Mijnendonckx et al., 2013; Recordati et al., 2021).

**Figure 1:**
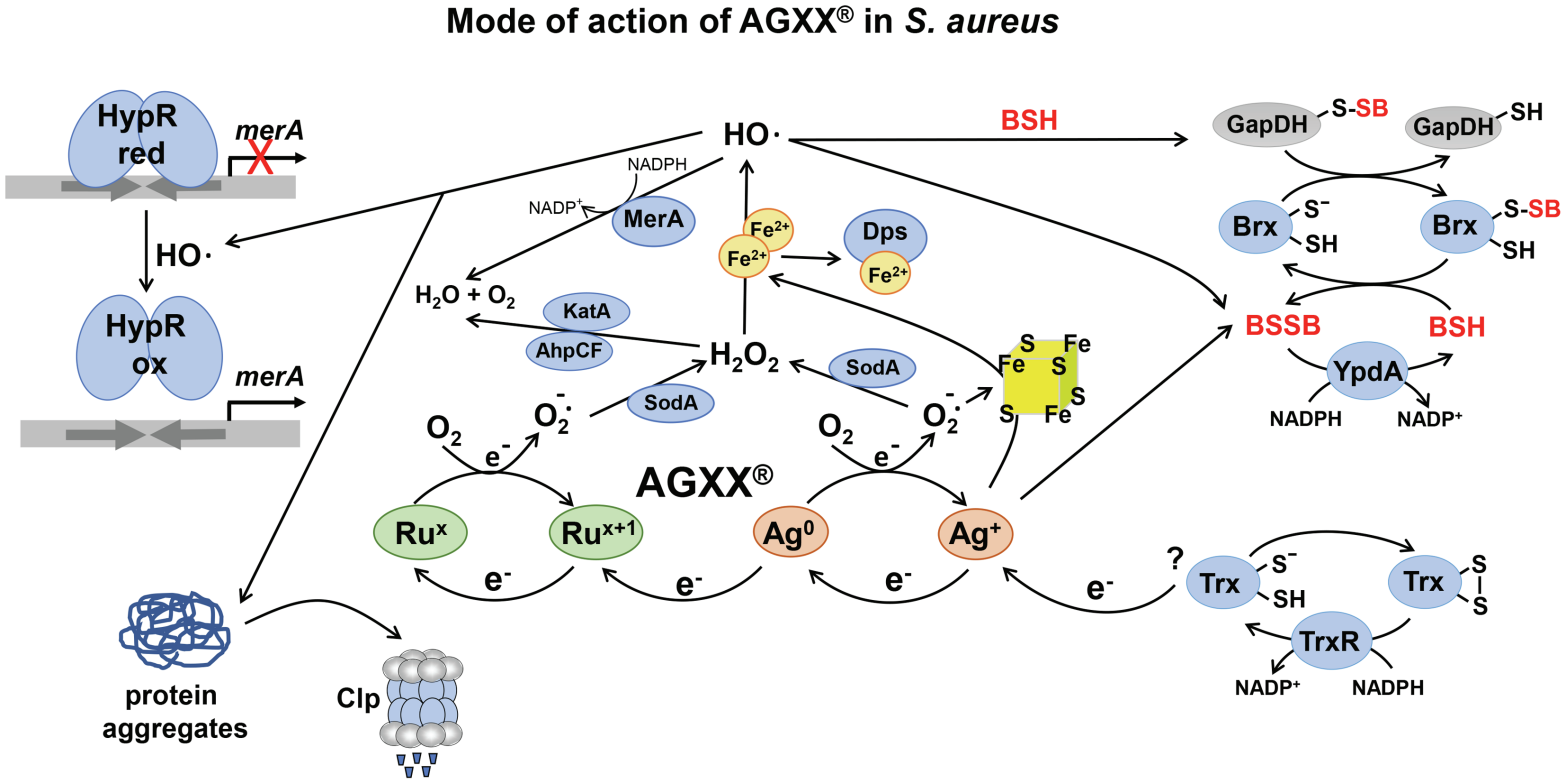
The proposed mode of action of AGXX® and the responses in *Staphylococcus aureus*. AGXX® is composed of silver (Ag^+^) and ruthenium (Ru^x^), which are connected by two redox cycles and form an electric field, leading to electron transfer to molecular oxygen (O_2_), with subsequent reactive oxygen species (ROS) generation, such as superoxide anion O_2_•^−^, hydrogen peroxide (H_2_O_2_), and hydroxyl radicals (OH•). First, Ag^0^ is oxidized to Ag^+^, which is back-reduced to Ag^0^ possibly by electrons from cellular donors, such as the thioredoxin (Trx)/thioredoxin reductase (TrxR) system. Second, Ru^x+1^ is reduced by electrons from Ag^0^, leading to formation of Ru^x^, which is re-oxidized to Ru^x+1^. ROS are generated in the AGXX® redox cycles upon oxidation of Ag^0^ and Ru^x^. Furthermore, Ag^+^ ions and O_2_•^−^ have been described to damage FeS clusters and release Fe^2+^, which potentiates OH• generation *via* the Fenton chemistry. In *S. aureus*, AGXX® induces antioxidant enzymes, such as catalases, peroxiredoxins, and superoxide dismutases for detoxification of ROS (Loi et al., 2018). AGXX® leads to thiol-oxidation of the HypR repressor, resulting in upregulation of the HypR-controlled flavin disulfide reductase MerA, which probably detoxifies OH•. In addition, AGXX® exposure resulted in increased protein *S*-bacillithiolation of GapDH and an oxidative shift of the BSH redox potential, supporting an impaired thiol-redox homeostasis. GapDH-SSB can be de-bacillithiolated in the Brx/BSH/YpdA redox pathway to regenerate GapDH. Consequently, BSH and MerA were shown to protect *S. aureus* against AGXX® toxicity (Loi et al., 2018). In addition, AGXX® causes oxidative protein unfolding and protein aggregates, which are degraded by the Clp protease complex. The figure is modified from (Guridi et al., 2015) including previous results from (Loi et al., 2018).

Recently, metal-based nanoenzymes, such as the oxidase-like silver-palladium bimetallic alloy nanocage AgPd_0.38_ were shown to produce ROS at the surface and selectively kill drug-resistant bacteria, but did not show toxicity in mammalian cells (Gao et al., 2021). In addition, very promising metal-based antimicrobial surface coatings were developed, including AGXX® and the combination of functionalized graphene oxide (GOX) with AGXX®, termed as GOX-AGXX® (Landau, 2013; Guridi et al., 2015; Vaishampayan et al., 2021). AGXX® is composed of Ag^+^ and ruthenium (Ru^+^), which form an electric field *via* two redox cycles, leading to electron transfer to molecular oxygen and ROS generation (Figure 1; Guridi et al., 2015; Clauss-Lendzian et al., 2018). In the first cycle, elementary Ag^0^ is oxidized to Ag^+^, which is regenerated by bacterial reducing pathways, such as the thioredoxin (Trx)/ thioredoxin reductase (TrxR) system. In the second cycle, higher valent Ru^x+1^ is reduced to Ru^x^, and re-oxidized to Ru^x+1^ with subsequent ROS formation, such as superoxide anion, H_2_O_2_, and the highly toxic OH•. These redox cycles lead to a constant regeneration and ensure longevity and sustainability of the antimicrobial metal coating (Landau, 2013; Guridi et al., 2015). Moreover, AGXX® is predicted to cause low toxicity in human cells, since it releases only small amounts of 0.1–0.2 mg/l of silver ions after 12 weeks of exposure in distilled water (Guridi et al., 2015), although detailed toxicity studies are lacking.

The novel combined GOX-AGXX® coating acts *via* a “catch and kill” mechanism to facilitate bacterial killing (Vaishampayan et al., 2021). GOXs are oxidized graphite sheets, which are grafted with polycationic polymer chains and bind the negatively-charged cell envelope of bacteria (Vaishampayan et al., 2021). Thus, GOX captures bacteria *via* electrostatic attractions, leading to inhibition of bacterial proliferation as bacteriostatic effect (Vaishampayan et al., 2021). The second material AGXX® kills the GOX-captured bacteria *via* the described ROS formation.

AGXX® can be electroplated on various materials, including plastics, steel, glass, ceramics, fleeze, and cellulose fibers. AGXX® coatings are used for sterilization of medical implants, catheters, and plasters as well as in industrial water pipelines to successfully eradicate bacteria, which are in close contact with the AGXX® surface by a process termed as “contact killing” (Maillard and Hartemann, 2013; Guridi et al., 2015; Villapún et al., 2016; Clauss-Lendzian et al., 2018; Vaishampayan et al., 2021).

AGXX® acts as promising broad-spectrum antimicrobial and revealed strong bactericidal effects against various multidrug resistant pathogens, such as *S. aureus*, *Staphylococcus epidermidis*, *Escherichia coli*, *Enterococcus faecalis*, *Enterococcus faecium*, and *Legionella erythra* (Landau, 2013; Guridi et al., 2015; Heiss et al., 2017; Clauss-Lendzian et al., 2018; Loi et al., 2018; Vaishampayan et al., 2018). In addition, AGXX® and GOX-AGXX® both inhibit biofilm formation, which was supported by the downregulation of virulence factor regulons (e.g., SaeRS and Agr) and genes for biofilm formation in *S. aureus* (Vaishampayan et al., 2018, 2021). RNAseq analyses further revealed that AGXX®373 causes an oxidative and metal stress response as well as proteotoxic effects in *S. aureus* (Loi et al., 2018). AGXX® treatment leads to protein-*S*-bacillithiolation of GapDH, increased protein aggregations and an oxidative shift in the bacillithiol (BSH) redox potential, supporting ROS generation as its main mode of action (Figure 1; Loi et al., 2018). We have further shown that the low molecular weight thiol BSH and the HypR-regulated flavin disulfide reductase MerA function in the defense against AGXX® in *S. aureus* (Loi et al., 2018; Linzner et al., 2021). Furthermore, the inductions of heat-shock specific proteases and chaperones and antioxidant responses were observed after AGXX® and GOX-AGXX® treatment in *E. faecium* and *S. aureus* (Clauss-Lendzian et al., 2018; Vaishampayan et al., 2021).

While global transcriptomic analyses revealed insights into the mode of action of these metal coatings in bacterial pathogens, the question arises how the efficiency can be improved for complete killing. Previous studies revealed that the antimicrobial activity of metal nanoparticles depends on the particle size (Raghupathi et al., 2011; Kadiyala et al., 2018). In our studies, we found that the two charges, AGXX®373 and AGXX®383, differ strongly in their antimicrobial activities, which correlates with their particle sizes and affects the efficient concentrations and times for *S. aureus* killing. While 20–40 μg/ml AGXX®373 with particle size of 1.5–2.5 μm kills *S. aureus* completely within 2 h, 2–3-fold higher amounts of 60–80 μg/ml AGXX®383 with particle size of >3.2 μm were required for the same extent of *S. aureus* killing. Thus, small AGXX® particles with a higher surface area are more efficient antimicrobials and kill bacteria at lower concentrations compared to larger size particles.

## Materials and Methods

### Preparation of AGXX® Microparticles

The AGXX®373 and AGXX®383 particles were produced by Largentec GmbH (Berlin, Germany) using different silver powders with the particle sizes of 1.5–2.5 μm (MaTecK, Germany) and >3.2 μm (Toyo, Japan) as described previously (Loi et al., 2018). Briefly, both silver powders were chemically coated with ruthenium in alkaline medium, where the Ru(III) ions were first oxidized by NaOCl to RuO_4_ (Guridi et al., 2015; Heiss et al., 2017). Reduction of RuO_4_ to Ru or RuO_X_ was performed by addition of NaNO_2_ as described (Chen et al., 2010). The black AGXX® powder was further conditioned with 50 mM ascorbic acid for 2 h, followed by filtration, washing with deionized water and drying.

### Bacterial Cultivation and Survival Assays

The multi-resistant *S. aureus* USA300JE2 strain (Fey et al., 2013) was used for the AGXX® survival experiments and cultivated in RPMI medium as described (Fritsch et al., 2020). For survival assays under AGXX® stress, *S. aureus* USA300JE2 was grown in RPMI medium to an optical density at 500 nm (OD_500_) of 0.5 and exposed to 10–40 μg/ml AGXX®373 and 40–100 μg/ml AGXX®383 microparticles as indicated in the figure legends. Ten microliter of serial dilutions of the cultures was spotted onto Luria Bertani (LB) agar plates and colony forming units (CFUs) monitored after overnight incubation at 37°C. For quantification, 100 μl of serial dilutions was plated onto LB agar plates and CFUs counted after overnight incubation. The survival rates were calculated of the treated cultures in comparison to the untreated control culture at an OD_500_ of 0.5. The survival of the untreated control was set to 100%.

## Results

The Smaller AGXX®373 Particles Show Higher Antimicrobial Activities Toward *S. aureus* USA300JE2 in Comparison to Larger Sized AGXX®383 Particles

Previously, we analyzed the oxidative mode of action of AGXX®373 in *S. aureus* using 5 μg/ml AGXX® as sub-lethal concentration, which inhibits the growth (Loi et al., 2018). In this study, we were interested in the applied aspects of the AGXX® coating. We investigated the time-course required for *S. aureus* killing upon challenge with the two different charges AGXX®373 and AGXX®383, which varied in their particle size. These AGXX®373 and AGXX®383 charges were generated from different silver powders obtained from the companies MaTeck (Germany) and Toyo (Japan), respectively. While AGXX®373 particles had a small particle size of 1.5–2.5 μm, the size of AGXX®383 particles was in the range of >3.2 μm.

**Figure 2:**
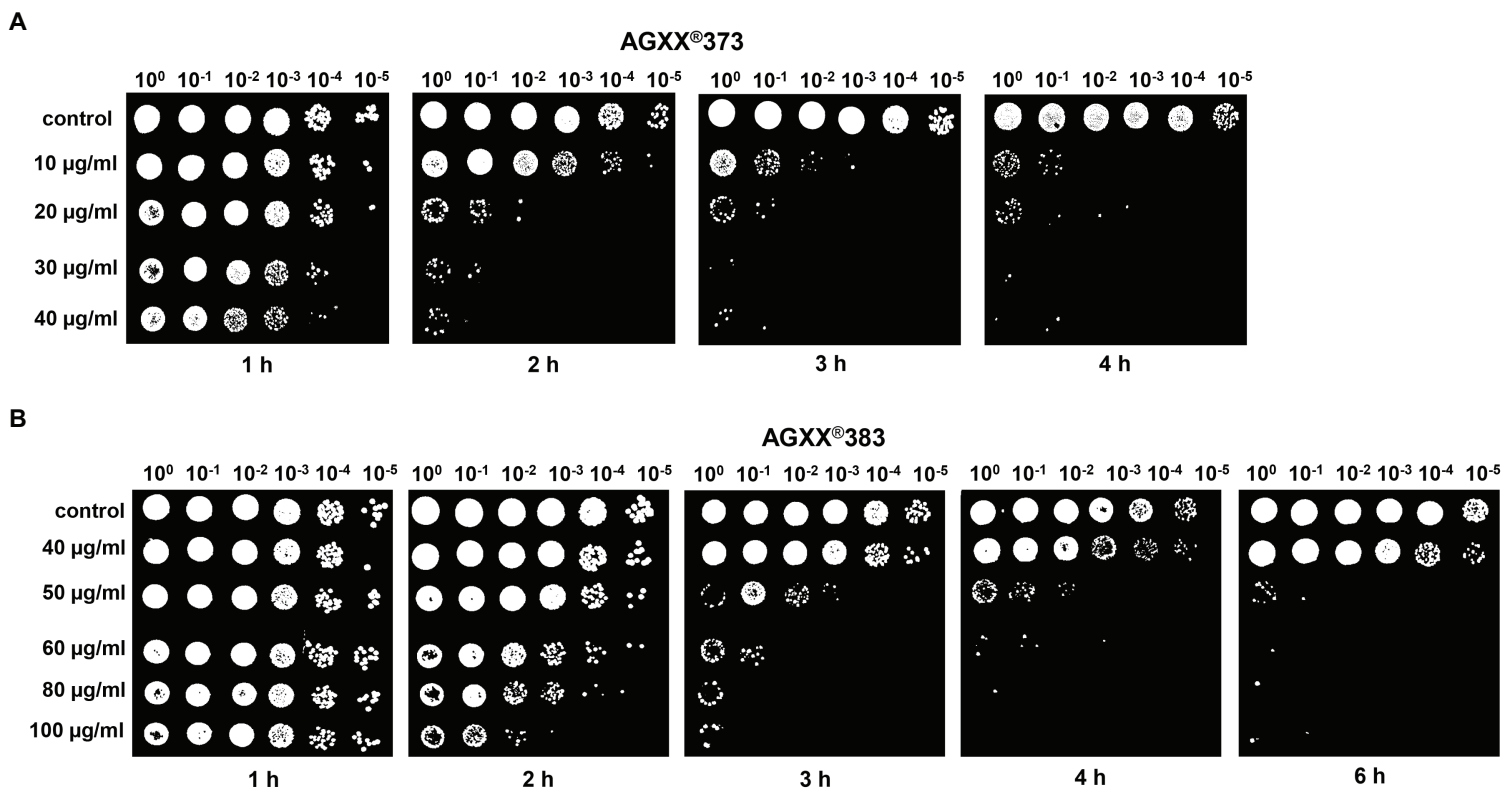
The charges AGXX®373 **(A)** and AGXX®383 **(B)** cause different antimicrobial effects in *S. aureus* USA300JE2. Survival assays of *S. aureus* USA300JE2 cells were performed at an OD_500_ of 0.5 after exposure to 10–40 μg/ml AGXX®373 **(A)** or 40–100 μg/ml AGXX® 383 **(B)**. Serial dilutions of the cultures were spotted after 1–6 h of AGXX® exposure onto Luria Bertani (LB) agar plates to observe colony forming units (CFUs) after 24 h incubation. The presented results are representatives of three biological replicates. The results indicate that AGXX®373 particles have a stronger killing effect compared to AGXX®383 particles in *S. aureus*.

**Figure 3:**
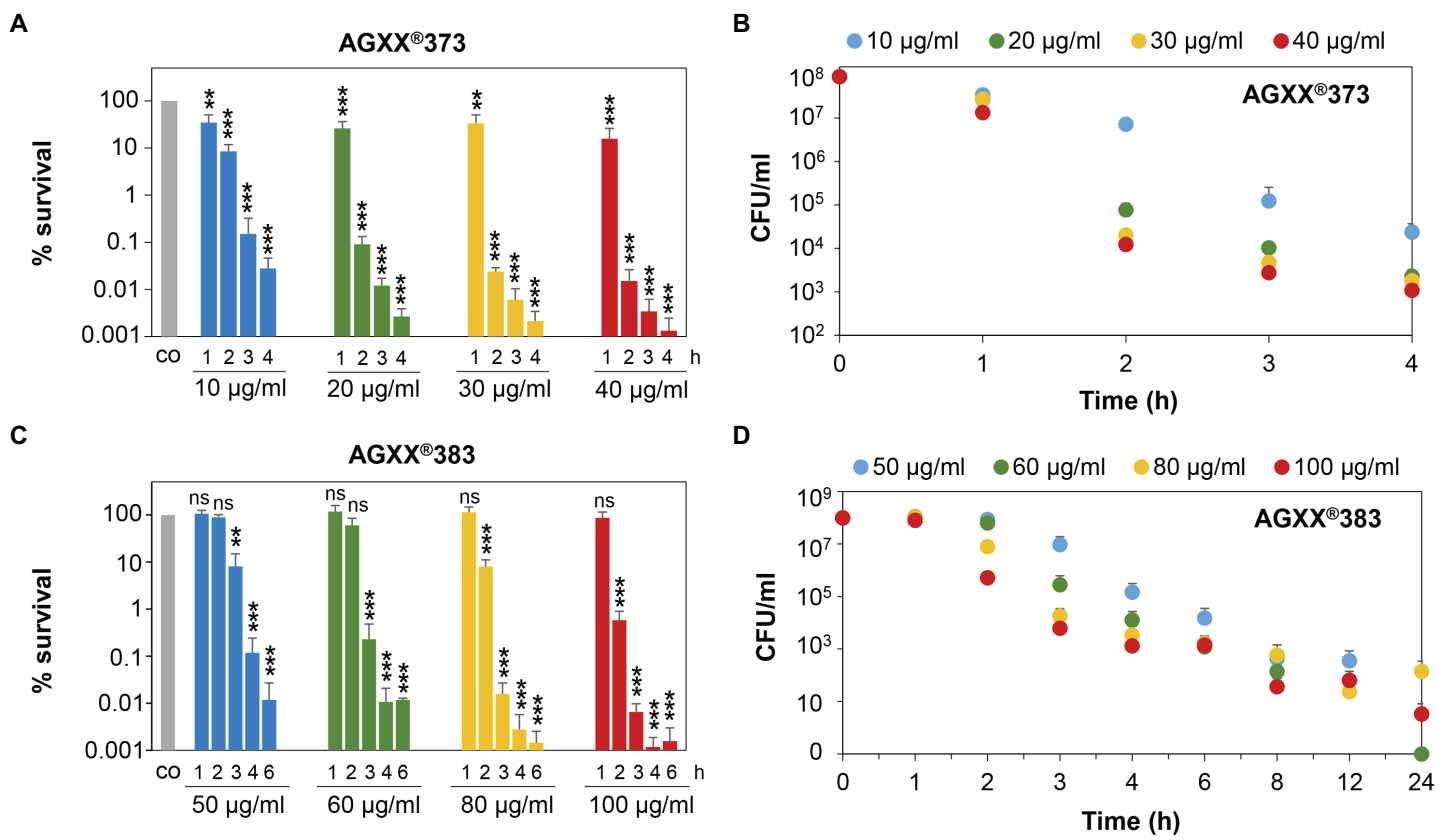
Survival assays indicate 2-3-fold increased killing efficiencies of smaller AGXX®373 versus larger AGXX®383 particles in *S. aureus*. *S. aureus* USA300JE2 was exposed to 10–40 μg/ml AGXX®373 particles **(A,B)** and 50–100 μg/ml AGXX®383 particles **(C,D)** at an OD_500_ of 0.5. After 1–24 h of AGXX® treatment, CFUs were determined from serial dilutions plated overnight on LB agar plates. The survival ratios of the CFUs after AGXX® stress were calculated relative to the untreated control (co), which was set to 100%. Mean values of 3–5 biological replicates are presented, and error bars represent the SD. For statistical analyses of the killing effect, the 100% survival of the untreated control (co) was compared to 10, 20, 30, and 40 μg/ml AGXX®373 **(A)** or 50, 60, 80, and 100 μg/ml AGXX®383 **(C)**, respectively, and the calculations were performed using a Student’s unpaired two-tailed *t*-test by the graph prism software. Symbols are *ns* > 0.05, ^**^p ≤ 0.01, and ^***^p ≤ 0.001.

To investigate the impact of the particle size and the exposure time, we performed various survival assays of *S. aureus* cultures exposed to 10–40 μg/ml of AGXX®373 and 40–100 μg/ml AGXX®383, respectively, in a time-dependent manner (Figures 2, 3). The spotted survival assays revealed that 20–40 μg/ml AGXX®373 were highly toxic, resulting in microbial killing after 2 h, while 50–100 μg/ml AGXX®383 were required to kill *S. aureus* to a similar extent within 3 h (Figures 2A,B). For comparison, a similar CFU reduction was observed after treatment of *S. aureus* with 30 μg/ml AGXX®373 for 2 h as with 60 μg/ml AGXX®383 for 3 h, pointing to a >2-fold increased efficiency of the smaller AGXX®373 particles. In addition, the *S. aureus* viability rate decreased continuously over time with both AGXX® charges, leading to almost complete killing after 4 h with 20–40 μg/ml AGXX®373 and 60–100 μg/ml AGXX®383 (Figures 2A,B). These data indicate that the AGXX® particle size and the exposure time affect significantly its antimicrobial activity.

Next, the survival rates of *S. aureus* cells were quantified after different times of exposure to AGXX®373 or AGXX®383 in more detail using CFU counting. In agreement with the above results, treatment of *S. aureus* with 20–40 μg/ml AGXX®373 resulted in a time-depending killing of 99.9% cells within 2 h and almost 99.999% of cells lost their viability after 4 h (Figures 3A,B; Supplementary Table S1). However, much higher concentrations of 60–100 μg/ml AGXX®383 were required to achieve a >99% killing efficiency after 3 h and >99.99% after 4 h (Figures 3C,D). While only 0.02% of *S. aureus* cells survived after 2 h exposure to 30 μg/ml AGXX®373, the survival was only slightly reduced to ~60% after 2 h treatment with 60 μg/ml AGXX®383 (Figures 3A,C). Furthermore, the decrease in bacterial viability was significant with 10–40 μg/ml AGXX®373 over the entire 1–4 h time course, whereas 50–60 μg/ml AGXX®383 did not show significant effects on *S. aureus* survival within 1–2 h. However, long-term exposure of *S. aureus* to 60–100 μg/ml AGXX®383 for 8–24 h led to a nearly 100% killing of cells (Figures 3C,D). Starting with a cell count of ~10^8^ of the log phase bacteria, the CFU dropped to ~10^3^ after 6 h, was further decreased to 23–63 colonies after 12 h and to 0–140 colonies after 24 h of exposure to 60–100 μg/ml AGXX®383 (Figure 3D; Supplementary Table S2). Importantly, almost complete killing of *S. aureus* was observed after exposure to 50–60 and 100 μg/ml AGXX®383, while the CFUs were slightly higher after treatment with 80 μg/ml AGXX®383 for unknown reasons.

In conclusion, our results confirmed that both AGXX®373 and AGXX®383 charges act strongly bactericidal and kill *S. aureus* USA300JE2 efficiently and nearly complete in a time- and dose-dependent manner. However, the concentrations and exposure times required for complete killing of *S. aureus* cultures were 2-3-fold higher for the larger size AGXX®383 particles compared to AGXX®373. Thus, the increased particle size of AGXX®383 led to a decreased antimicrobial activity compared to the smaller AGXX®373 particles. Taken together, our results indicate that a low particle size and long exposure time are crucial for maximal killing efficiency of the AGXX® powder.

## Discussion

In the present study, we have quantified the killing effect of different particles sizes of the AGXX® antimicrobial surface coating in *S. aureus*. We used the smaller AGXX®373 and larger AGXX®383 particle charges to analyze time-dependent killing effects in survival assays. Our results showed that both AGXX® particles had strong antimicrobial activities, which were dependent on the particle size, exposure time, and the concentration. Overall, the antimicrobial efficiency of AGXX® particles was most efficient with high concentrations, long exposure times, and smaller particles sizes. In comparison, we would suggest to use charge AGXX®373 for future applications, due to its small particle size of 1.5–2.5 μm and its strong killing effect with low concentrations of 20–40 μg/ml. Much higher concentrations of 60–100 μg/ml were required to obtain the same killing efficiency with AGXX®383, which had a particle size of ~3.4 μm. Thus, the particle size has a strong impact on the antimicrobial activity and efficiency of the AGXX® coating. Similar connections between the particle size and the antimicrobial activity were previously obtained for other metal nanoparticles, such as 4,6-diamino-2-pyrimidine thiol (DAPT)-capped gold nanoparticles, zinc oxide, and silver nanoparticles (Raghupathi et al., 2011; Lu et al., 2013; Zheng et al., 2021). The antimicrobial activities of the larger gold nanoparticles with the size of >5.5 nm toward *E. coli*, *Pseudomonas aeruginosa*, and *Klebsiella pneumoniae* were significantly lower compared to nanoparticles of 1.8–5.5 nm (Zheng et al., 2021). Furthermore, the antibacterial activities of smaller zinc oxide and silver nanoparticles were higher against *S. aureus* and *Streptococcus mutans*, respectively, compared to larger size nanoparticles (Raghupathi et al., 2011; Lu et al., 2013). The impact of the particle size on the antimicrobial activity can be explained by the higher surface/volume ratio of smaller particles, which provide an increased surface for “contact killing” of microbes by the metal coating. The term “contact killing” indicates that bacteria and yeast cells are rapidly killed on metallic surfaces, such as copper and silver alloys by their close contact with the metal surface, causing damage of the cellular envelope and macromolecules (Grass et al., 2011; Quaranta et al., 2011). The probability that bacteria come in close contact with the metal particles increases with a larger surface/volume ratio, leading to an increased antimicrobial activity (Kadiyala et al., 2018). Thus, our results support previous data on other metal nanoparticles and their antimicrobial activity, which strongly depend on the particle size. Given the wide range of applications of the AGXX® surface coating in medicine, agriculture, and industries, it is very important to keep the size of metal particles as low as possible and reproducible to achieve efficient antimicrobial activities.

## Data Availability Statement

The original contributions presented in the study are included in the article/Supplementary Material; further inquiries can be directed to the corresponding author.

## Author Contributions

NL designed and performed the experiments, analyzed the data, and wrote the manuscript draft. NL and HA revised and approved the manuscript. All authors contributed to the article and approved the submitted version.

## Conflict of Interest

The authors declare that the research was conducted in the absence of any commercial or financial relationships that could be construed as a potential conflict of interest.

## Publisher’s Note

All claims expressed in this article are solely those of the authors and do not necessarily represent those of their affiliated organizations, or those of the publisher, the editors and the reviewers. Any product that may be evaluated in this article, or claim that may be made by its manufacturer, is not guaranteed or endorsed by the publisher.
