# Supplementary material for: The Antimicrobial Activity of the AGXX® Surface Coating Requires a Small Particle Size to Efficiently Kill Staphylococcus aureus
Source: Front Microbiol. 2021 Aug 12;12:731564. doi: 10.3389/fmicb.2021.731564 (PMC8387631; doi:10.3389/fmicb.2021.731564)
Supplement: Supplementary file 1 [file Data_Sheet_1.PDF]

Table S1. CFUs and survival rates of *S. aureus* USA300JE2 after exposure to 10-40 µg/ml AGXX®373 for 1-4 h

**Time (h)**	**10 µg/ml AGXX®373**	
	**CFU/ml**	**Survival rate (%)**
**0**	8.94×10^{7}	100.00
**1**	$3.40 \times 10^{7} \pm 1.09 \times 10^{7}$	34.48 ± 16.20
**2**	$7.20 \times 10^{6} \pm 2.28 \times 10^{6}$	8.56 ± 3.31
**3**	$1.22 \times 10^{5} \pm 1.32 \times 10^{5}$	0.15 ± 0.18
**4**	$2.37 \times 10^{4} \pm 1.34 \times 10^{4}$	0.028 ± 0.018
**Time (h)**	**20 µg/ml AGXX®373**	
	**CFU/ml**	**Survival rate (%)**
**0**	8.94×10^{7}	100.00
**1**	$2.63 \times 10^{7} \pm 5.62 \times 10^{6}$	26.05 ± 10.41
**2**	$7.70 \times 10^{4} \pm 3.20 \times 10^{4}$	0.091 ± 0.042
**3**	$1.03 \times 10^{4} \pm 2.94 \times 10^{3}$	0.012 ± 0.0052
**4**	$2.27 \times 10^{3} \pm 1.02 \times 10^{3}$	0.0027 ± 0.0012
**Time (h)**	**30 µg/ml AGXX®373**	
	**CFU/ml**	**Survival rate (%)**
**0**	8.94×10^{7}	100.00
**1**	$2.71 \times 10^{7} \pm 1.07 \times 10^{6}$	33.55 ± 16.95
**2**	$2.03 \times 10^{4} \pm 1.82 \times 10^{3}$	0.024 ± 0.0050
**3**	$4.76 \times 10^{3} \pm 3.17 \times 10^{3}$	0.0060 ± 0.0043
**4**	$1.78 \times 10^{3} \pm 9.28 \times 10^{2}$	0.0021 ± 0.0013
**Time (h)**	**40 µg/ml AGXX®373**	
	**CFU/ml**	**Survival rate (%)**
**0**	8.94×10^{7}	100.00
**1**	$1.33 \times 10^{7} \pm 7.79 \times 10^{6}$	15.73 ± 10.39
**2**	$1.23 \times 10^{4} \pm 8.90 \times 10^{3}$	0.015 ± 0.011
**3**	$2.77 \times 10^{3} \pm 2.14 \times 10^{3}$	0.0034 ± 0.0028
**4**	$1.08 \times 10^{3} \pm 8.74 \times 10^{2}$	0.0013 ± 0.0012

Table S2. CFUs and survival rates of *S. aureus* USA300JE2 after exposure to 50-100 µg/ml AGXX®383 for 1-24 h

Time (h)	50 µg/ml AGXX®383	
	CFU/ml	Survival rate (%)
**0**	9.96×10^7	100.00
**1**	$1.01 \times 10^8 \pm 3.30 \times 10^6$	105.62 ± 19.42
**2**	$8.60 \times 10^7 \pm 1.16 \times 10^7$	88.11 ± 12.95
**3**	$9.39 \times 10^6 \pm 9.39 \times 10^6$	8.06 ± 6.88
**4**	$1.44 \times 10^5 \pm 1.66 \times 10^5$	0.12 ± 0.12
**6**	$1.50 \times 10^4 \pm 1.98 \times 10^4$	0.012 ± 0.015
**8**	$4.33 \times 10^2 \pm 4.93 \times 10^2$	0.00036 ± 0.00037
**12**	$3.50 \times 10^2 \pm 4.88 \times 10^2$	0.00027 ± 0.00037
**24**	3 ± 5	0.0000026 ± 0.0000037
**Time (h)**	**60 µg/ml AGXX®383**	
	**CFU/ml**	**Survival rate (%)**
**0**	9.96×10^7	100.00
**1**	$1.11 \times 10^8 \pm 2.19 \times 10^7$	118.12 ± 39.22
**2**	$6.38 \times 10^7 \pm 3.60 \times 10^7$	60.14 ± 24.71
**3**	$2.80 \times 10^5 \pm 3.37 \times 10^5$	0.23 ± 0.25
**4**	$1.28 \times 10^4 \pm 1.36 \times 10^4$	0.011 ± 0.010
**6**	$1.21 \times 10^3 \pm 1.32 \times 10^3$	0.0010 ± 0.00098
**8**	$1.40 \times 10^2 \pm 1.14 \times 10^2$	0.00014 ± 0.00014
**12**	63 ± 76	0.000071 ± 0.000090
**24**	0	0
**Time (h)**	**80 µg/ml AGXX®383**	
	**CFU/ml**	**Survival rate (%)**
**0**	9.96×10^7	100.00
**1**	$1.06 \times 10^8 \pm 1.82 \times 10^7$	112.85 ± 34.81
**2**	$8.02 \times 10^6 \pm 3.35 \times 10^6$	7.98 ± 3.10
**3**	$1.82 \times 10^4 \pm 1.61 \times 10^4$	0.016 ± 0.011
**4**	$3.39 \times 10^3 \pm 3.97 \times 10^3$	0.0028 ± 0.0030
**6**	$1.67 \times 10^3 \pm 1.50 \times 10^3$	0.0015 ± 0.0011
**8**	$5.93 \times 10^2 \pm 8.39 \times 10^2$	0.00046 ± 0.00065
**12**	23 ± 33	0.000018 ± 0.000026
**24**	$1.40 \times 10^2 \pm 1.98 \times 10^2$	0.00011 ± 0.00015
**Time (h)**	**100 µg/ml AGXX®383**	
	**CFU/ml**	**Survival rate (%)**
**0**	9.96×10^7	100.00
**1**	$8.10 \times 10^7 \pm 1.51 \times 10^7$	86.78 ± 28.17
**2**	$5.20 \times 10^5 \pm 2.29 \times 10^5$	0.58 ± 0.31
**3**	$6.17 \times 10^3 \pm 2.46 \times 10^3$	0.0066 ± 0.0033
**4**	$1.32 \times 10^3 \pm 9.94 \times 10^2$	0.0012 ± 0.00068
**6**	$1.41 \times 10^3 \pm 1.19 \times 10^3$	0.0016 ± 0.0014
**8**	37 ± 39	0.000041 ± 0.000047
**12**	63 ± 76	0.000071 ± 0.000090
**24**	3 ± 5	0.0000040 ± 0.0000056
